# The response of *Sphingopyxis granuli* strain TFA to the hostile anoxic condition

**DOI:** 10.1038/s41598-019-42768-9

**Published:** 2019-04-18

**Authors:** Yolanda Elisabet González-Flores, Rubén de Dios, Francisca Reyes-Ramírez, Eduardo Santero

**Affiliations:** Centro Andaluz de Biología del Desarrollo/CSIC/Universidad Pablo de Olavide/Junta de Andalucía. Departamento de Biología Molecular e Ingeniería Bioquímica, Seville, Spain

**Keywords:** Bacteria, Microbial genetics, Bacteria, Microbial genetics

## Abstract

Sphingomonads comprises a group of interesting aerobic bacteria because of their ubiquity and metabolic capability of degrading many recalcitrant contaminants. The tetralin-degrader *Sphingopyxis granuli* strain TFA has been recently reported as able to anaerobically grow using nitrate as the alternative electron acceptor and so far is the only bacterium with this ability within the sphingomonads group. To understand how strain TFA thrives under anoxic conditions, a differential transcriptomic analysis while growing under aerobic or anoxic conditions was performed. This analysis has been validated and complemented with transcription kinetics of representative genes of different functional categories. Results show an extensive change of the expression pattern of this strain in the different conditions. Consistently, the most induced operon in anoxia codes for proteases, presumably required for extensive changes in the protein profile. Besides genes that respond to lack of oxygen in other bacteria, there are a number of genes that respond to stress or to damage of macromolecules, including genes of the SOS DNA-damage response, which suggest that anoxic conditions represent a hostile environment for this bacterium. Interestingly, growth under anoxic conditions also resulted in repression of all flagellar and type IV pilin genes, which suggested that this strain shaves its appendages off while growing in anaerobiosis.

## Introduction

Bacteria of the sphingomonads group belong to the physiologically diverse *Sphingomonadaceae* Family, within the Class α-proteobacteria, and were originally clustered within the genus *Sphingomonas*. However, phylogenetic analyses of the new described species in the past two decades have shown the relative genetic diversity of the species, which can be classified into at least 5 genera, *Sphingomonas, Novosphingobium, Sphingopyxis* and *Sphingobium*^1^ and, more recently, *Sphingosinicella*^2^. This group of bacteria have attracted attention because of its ubiquity -since they can be isolated from many different environments, although many of them thrive in marine environments-their biodiversity, and their metabolic versatility. Analyses of their genomes showed that they are quite diverse in size, ranging from 3 Mb of *S. baekryungensis* DSM 16222 to 5 Mb of *S. fribergensis* Kp5.2, and in organization^3^. In addition, they have great plasticity, showing evidences for horizontal transfer of genomic islands, prophages, an even transposons, as the kanamycin resistance gene from a Tn903 transposon in a *Sphingobium* isolate, in their genomes^4,5^. A number of sphingomonads have been described as oligotrophic bacteria^6^, some of which play an important role in marine environments^7^, and a number of genes for marine adaptation and quorum sensing have been identified^8^. From an environmental point of view, sphingomonads show a high capacity of adaptation to stressing environments such as those rich in metals^9^, and a great metabolic versatility, being many strains of this group reported as able to metabolise many different compounds recalcitrant to microbial degradation such as polyaromatic hydrocarbons from crude oils^10^ or different xenobiotic compounds.

Most of the 11 *Sphingopyxis* species whose genome has been sequenced are strains with a reported degrading capability of recalcitrant or xenobiotic compounds such as polyethylene or polypropylene glycols, lindane, tetralin, phthalate, phenyl acetate or styrene. Within these, strain TFA is the only strain in which tetralin biodegradation has been fully characterised.

Strain TFA was isolated from the river Rhine sediments after selection for growth on tetralin and is one of the few strains reported to grow on tetralin as the only carbon and energy source^11^. The tetralin biodegradation pathway has been completely elucidated at biochemical and genetic levels by López-Sanchez *et al*.^12^ (and references therein) and the complex regulation of the *thn* operons, in which three regulatory systems are involved characterised by García-Romero *et al*.^13^ (and references therein). The genome of strain TFA was recently sequenced and the strain definitely ascribed to *Sphingopyxis granuli*. Its genome shows a number of genomic islands, integrated plasmid genes and prophages indicative of large genome plasticity. It also shows characteristics of oligotrophic bacteria and biodegradation genes besides those of the tetralin biodegradation pathway, which might confer additional uncharacterised biodegrading capabilities to this strain^4^.

Although sphingomonads have been described as strict aerobic bacteria^1^, the TFA genome showed a cluster of genes potentially involved in nitrate respiration and a *norB* gene coding for a nitric oxide reductase. Consistently, TFA was shown to grow anaerobically if nitrate is provided in the culture medium^4^. Although nitrate reduction to nitrite has also been reported in two other *Sphingopyxis* strains^14,15^, this reduction has not been characterised at all and anaerobic growth has not been reported for these strains, thus TFA represents the only strain within the sphingomonads group able to anaerobically grow using nitrate as the electron acceptor.

However, genes coding for nitrite reductase or nitrous oxide reductase, required for complete denitrification could not be found in the TFA genome. In agreement with this, anaerobic growth of strain TFA resulted in progressive reduction of nitrate levels and an almost stoichiometric accumulation of nitrite, which suggested that nitrite could not be respired (Supplementary Fig. S1). Actually, anaerobic growth was limited by nitrite accumulation, which fully restrained growth above a toxic concentration of 20 mM^4^, thus suggesting that growth of TFA under anoxic conditions may represent a hostile environment to the bacterium.

In order to characterise how the sphingomonad strain TFA thrives under anoxic conditions, we have compared the transcriptional gene expression pattern of this strain under aerobic and anoxic conditions with nitrate as the alternative electron acceptor. Global differential transcriptomic analyses together with transcriptional gene regulation kinetics showed that, in addition to the conventional genes induced by lack of oxygen in other bacteria, TFA regulated a number of other genes that are presumably involved in response to stress conditions and genes involved in damage repair, including the SOS DNA-damage response, thus indicating that this condition is hostile to the bacterium.

## Results

In order to characterise the global regulation during anaerobiosis of *S. granuli* strain TFA and to establish what genes could be directly regulated by anaerobiosis or by other conditions intrinsic to limited growth of this bacterium^8^, such as slow growth, nutrient or energy limitation or responses to other forms of stress, we compared the transcriptomic profiles by differential RNA-Seq in a number of conditions including aerobic growth on β-hydroxybutirate (BHB), which is its conventional carbon source, or on tetralin as the only carbon and energy sources, growth on BHB under anoxic conditions while respiring nitrate, and aerobic response to nitric oxide while growing on BHB (for specific conditions, see Methods). Transcription levels of selected differentially regulated genes under anaerobiosis belonging to different functional categories were subsequently estimated by RT-qPCR to confirm the global data and to obtain a kinetic of the transcriptional response.

**Figure 1:**
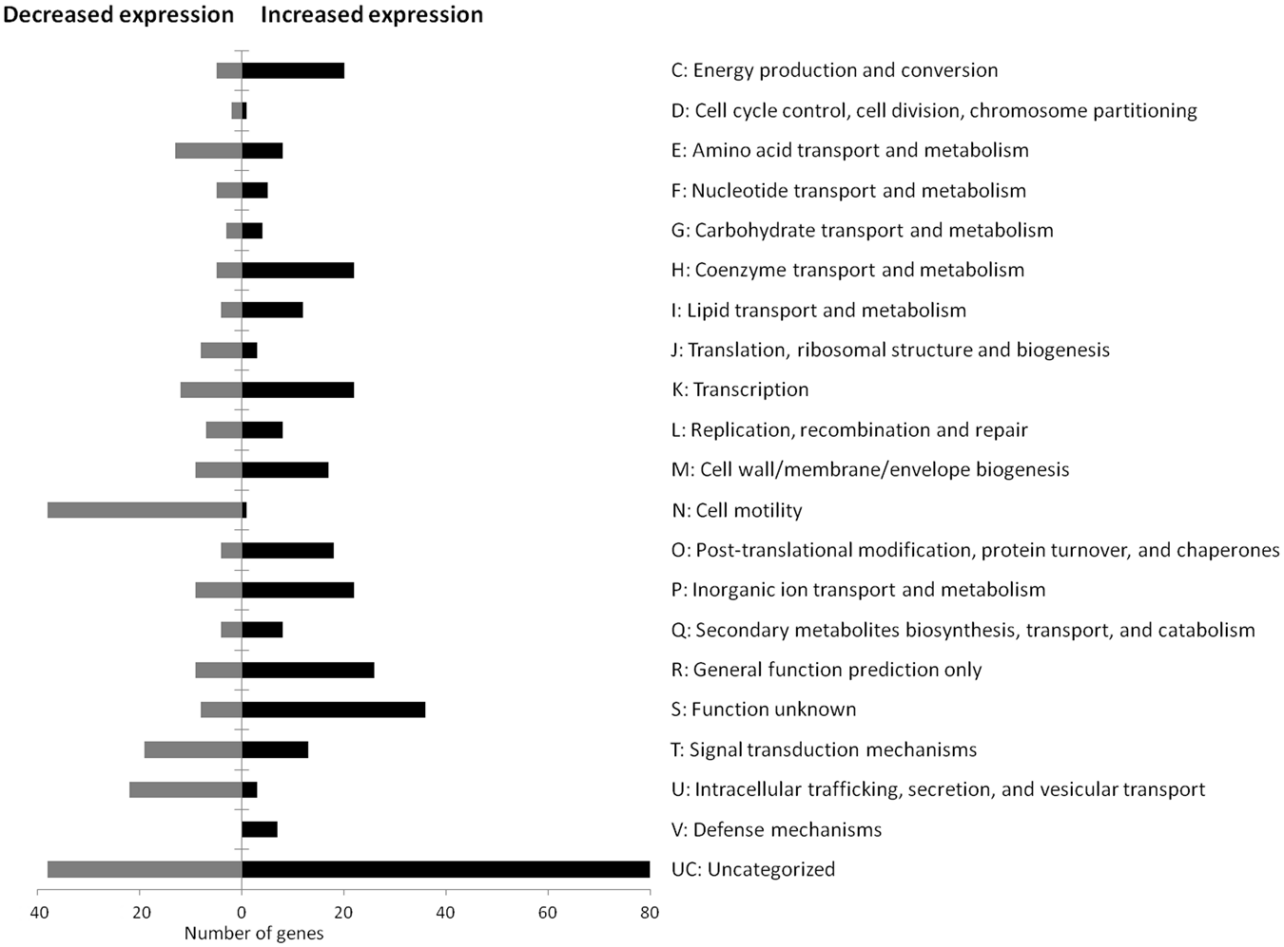
Distribution of anaerobically regulated genes of *S. granulli* strain TFA in COG categories. Genes represented here showed a differential expression of at least 3-fold.

Differential expression represented as fold induction or repression of each gene in the wild type strain while growing in (i) anoxic condition with nitrate using BHB as the carbon and energy source, (ii) in aerobiosis using tetralin as the carbon and energy source, and (iii) in aerobiosis using BHB as the carbon and energy source in the presence of nitric oxide, each of these conditions compared to the condition aerobic growth using BHB as the carbon and energy source, is shown in Supplementary Table S1. Growth in anaerobiosis resulted in an extensive change of the expression profile as compared to that while growing aerobically in the same medium. A total of 586 genes showed a differential transcription of at least 3-fold between the two conditions, 366 of them being induced whilst 220 being repressed during anaerobic growth. The number of genes belonging to each functional COG categories is shown in Fig. 1 (see Supplementary Table S1 for genes identification). Many differentially transcribed genes were not included in any of the functionally defined COG categories because they code for uncharacterized proteins or belong to the general function prediction only or to the function unknown category. However, by studying the genes belonging to defined functional categories a number of general features while growing in anaerobiosis could be observed.

In general, most of the genes coding for ribosomal proteins and other related factors such as elongation factors G, Tu, Ts and EF-4, and the ribosome recycling factor *frr* were repressed 2 to 4-fold, although only those differentially transcribed ≥3-fold are shown in Fig. 1 (for details, see Supplementary Table S1). On the other hand, one of the 3 genes coding for the translation initiation factor-1 (*infA*, SGRAN_2506) was induced under anaerobic conditions. Differential expression of these genes is probably a consequence of the lower growth rate under anaerobic conditions since all these genes were also differentially regulated to similar or even higher extent by slow growth conditions in aerobiosis, such as aerobic growth using tetralin as the carbon source (Supplementary Table S1). However, the contiguous genes coding for the ribosomal silencing factor RsfS (SGRAN_0786) and the 23 S rRNA methyltransferase RlmH (SGRAN_0785) were specifically induced by anaerobiosis 4-fold and not by aerobic slow growth conditions (Supplementary Table S1), which suggests that the ribosomes are specifically modified in response to anaerobic conditions.

From a metabolic point of view, cells growing in anaerobiosis do not seem to be greatly altered since the number of differentially expressed genes in categories E, F and G is not high and in most instances, the fold change is low. On the other hand, a high number of genes included in energy production and conversion was differentially transcribed but almost all of these genes code for electron transport components of the respiratory chains (see below).

A substantial number of genes (42) related to lipid transport and metabolism (I) and cell wall/membrane/envelope biogenesis (M), which include a number of transporters, polysaccharide biosynthesis proteins and cell wall modifying proteins such as lytic transglycosylases and 2 of the 3 cellulose biosynthesis proteins were differentially expressed in anaerobiosis. This suggests that the lipid, saccharide and protein components of the cell wall and membranes may be substantially different during growth in anaerobiosis. Differences in membrane-associated protein composition may actually be even greater because most of the differentially transcribed genes in P and V COG categories actually are components of transport systems.

It is also noteworthy the large number of genes of the cell motility category (N) that were repressed. All these genes are flagellar, pilin and chemotaxis genes. Additional flagellar and pilin genes that are assigned to the intracellular trafficking, secretion, and vesicular transport COG category (U), were also repressed in anaerobiosis. Additional chemotaxis genes repressed in anaerobiosis are assigned to the signal transduction COG category (T).

Regarding post-translational modification, protein turnover, and chaperones (COG code O), two proteases and one chaperone were induced in anaerobiosis but most of the induced genes assigned to this category actually code for proteins somehow related to stress/detoxification/repair such as peroxiredoxins (AhpC and AhpC2), thioredoxins, the methionine sulfoxide reductase MsrB, sulfide:quinone reductase and the SufABCD proteins (Fe-S clusters biogenesis), RadA DNA repair protein, and 1 Glutathione S-transferase-like protein (see below).

Finally, 38 genes coding for transcription factors including sigma factors, sensors and transcriptional regulators assigned to transcription (K) and signal transduction (T) COG categories were also differentially regulated ≥3-fold and probably are the responsible for different levels of regulation that expand the global response to anaerobiosis. Additionally, SGRAN_1132, coding for the global translational repressor CsrA (RsmA)^16^, was induced 6-fold.

A selection of differentially transcribed genes with a putative defined function derived from its similarity to known genes in the database are shown in Table 1 together with their fold regulation under anaerobic conditions and aerobic conditions in the presence of nitric oxide. In an attempt to gain more insights into the physiological situation under anaerobic conditions and the S. *granuli* response to this condition, 18 genes with different potential functions were selected to confirm their regulation by anaerobiosis and to characterise their induction kinetics under anaerobic and other stress conditions by RT-qPCR.

**Table 1:** Selected genes belonging to different functional categories that are differentially expressed in anaerobiosis.

SGRAN_id^a^	Gene name or product description	Fold regulation	
		−O_2_^b^	NO^c^
**Replication, cell division and control**			
3324	*ctrA*	−3.5	≤3
2088	*ccrM*	−7	≤3
3445–3443	ParA3-unknown-*ftsN*	−4 to −6	≤3
2409	*ftsB*	−3.5	≤3
1482	*ftsW*	−3	≤3
2978	*ndk* nucleoside diphosphate kinase	−5	≤3
3862	*nrdZ*	70	≤3
**Respiratory electron transfer chains**			
0222	*moeB*	20	≤3
3846–3856	*narGHJInifMmoaADEBCmoeA*	20 to 40	≤3
3857	*mobA* (convergent with *nar* operon)	8	≤3
2997–2991	*cyoABCDsurF1regBA*	7 to 48	2.5 to 9
0616–0615	*cydAB*	6 to 8	≤3
0617–0618	*cydDC*	5	≤3
2449–2452	*ccoNOQPG*	≤2,8	−3 to −11
2454–2456	*ccoHIS*	4.5 to 7	≤3
1792	*Aox*	5	5
**Protein turnover**			
3859–3860	*yhbUV*	98 to 360	≤3
**Stress response/detoxification**			
0384	*lsfA* Peroxidase	13	≤3
0624	*ahpC* Peroxiredoxin	5	≤3
0625	trxC thioredoxin	4	≤3
0626	*ahpC2* Peroxiredoxin	9	≤3
3865	Cytochrome-c peroxidase	6	≤3
2345–2348	*ectABCD*	8 to 10	≤3
0888	General stress protein	3.5	≤3
0590	Universal stress family protein 3	4	≤3
3353	Universal stress protein UspA	3	≤3
1161	ECF sigma factor	26	≤3
0596–0593	Sulfur detoxification and transport, and alkylhydroperoxide reductase	4 to 22	≤3
0597–0601	Sulfur detoxification and export	4 to 17	≤3
3840–3842	Sulfur detoxification and export	9 to 11	5
1321–1324	*arsR3H2C3B2*	6	9
3802–3800	Cupin-*norB -nnrS2*	5 to 14	4 to 18
3394–3396	Protein transport across the membrane	120 to 210	142 to 229
3393	*nsrR* (NO-sensitive regulator)	7	5
1632	*ytfE* (Fe-S clusters repair protein)	143	213
1371–1365	SUF (Fe-S clusters synthesis and assembly)	3 to 5	7 to 13
**SOS DNA repair system**			
1944	*lexA*	6	≤3
2940	*recA*	6	≤3
3642	*rada*	5	≤3
3044	*dinG*	4	≤3
3201–3199	*imuABdnaE2*	11 to 12	≤3
4085–4087	*imuA2B2dnaE2.1*	10	≤3
3204–3205	SRAP domain containing protein and *alkB*	8 to 10	≤3
2555–2550	SRAP domain containing proteins	8 to 10	≤3
**Bacterial appendages, motility and adherence**			
4088	*fliC*	−19	≤3
4089	*fliC2*	−23	≤3
4090	*fliE*	−11	≤3
4105	*fliD*	−7	≤3
4122	*flgM*	−14	≤3
4109	*motA*	−13	≤3
2956–2952	*cheAWYBR*	−5 to −11	≤3
3155	Methyl-accepting chemotaxis protein	−9	≤3
1907–1901	*cpaABCDEtadBC* (Pilus assembly proteins)	−4 to −13	≤3
3632	Flp/Fap pilin component	−9	≤3
0765–0767	*tadGE1E2*	−4 to −5	≤3
3043	*pilZ* (Type IV pilus assembly proteins)	−9	≤3
3775	*cpaF*	−5	≤3

(a) Gene number _id of *S. granuli*. Decreasing numbering in operons indicates that the operon is coded in the minus strand. (b) Ratio of values in anaerobiosis/aerobiosis for induced genes in anaerobiosis (shown as positive values) or the inverse ratio for repressed genes (shown as negative values). (c) Ratio of values in aerobiosis after adding NO/aerobiosis, for induced genes in response to NO (shown as positive values) or the inverse ratio for repressed genes (shown as negative values).

### Respiratory electron transfer chains

Expression of genes coding for the conventional aerobic cytochrome *c* oxidase *aa*3 were not substantially altered (less than 2-fold). However, the annotated genome indicated that strain TFA has up to 5 alternative terminal electron acceptors. These are the cytochrome *c* oxidase *cbb*_3_, encoded by the *cco* genes, the quinol oxidases *bo*_3_ and *bd*, encoded by the *cyo* and *cyd* genes, respectively, and the alternative oxidase *aox*, which transfer electrons to oxygen, and the respiratory nitrate reductase.

The genes coding for a putative nitrate/nitrite transport protein, the respiratory nitrate reductase components and proteins involved in biogenesis of the molibdenum cofactor are clustered together in the *narGHJInifMmoaADEBCmoeA* operon, comprising 12 genes (SGRAN_3845-3856) that are very closely located or even overlapped, thus suggesting translational coupling among many of them. They were induced 20- to 40-fold in anaerobiosis. *mobA* (SGRAN_3857) and *moeB* (SGRAN_0222) genes, presumably involved in the nitrate reductase Mo-cofactor biosynthesis but located outside this operon, were also induced by anaerobiosis 8 and 20-fold, respectively (Table 1).

**Figure 2:**
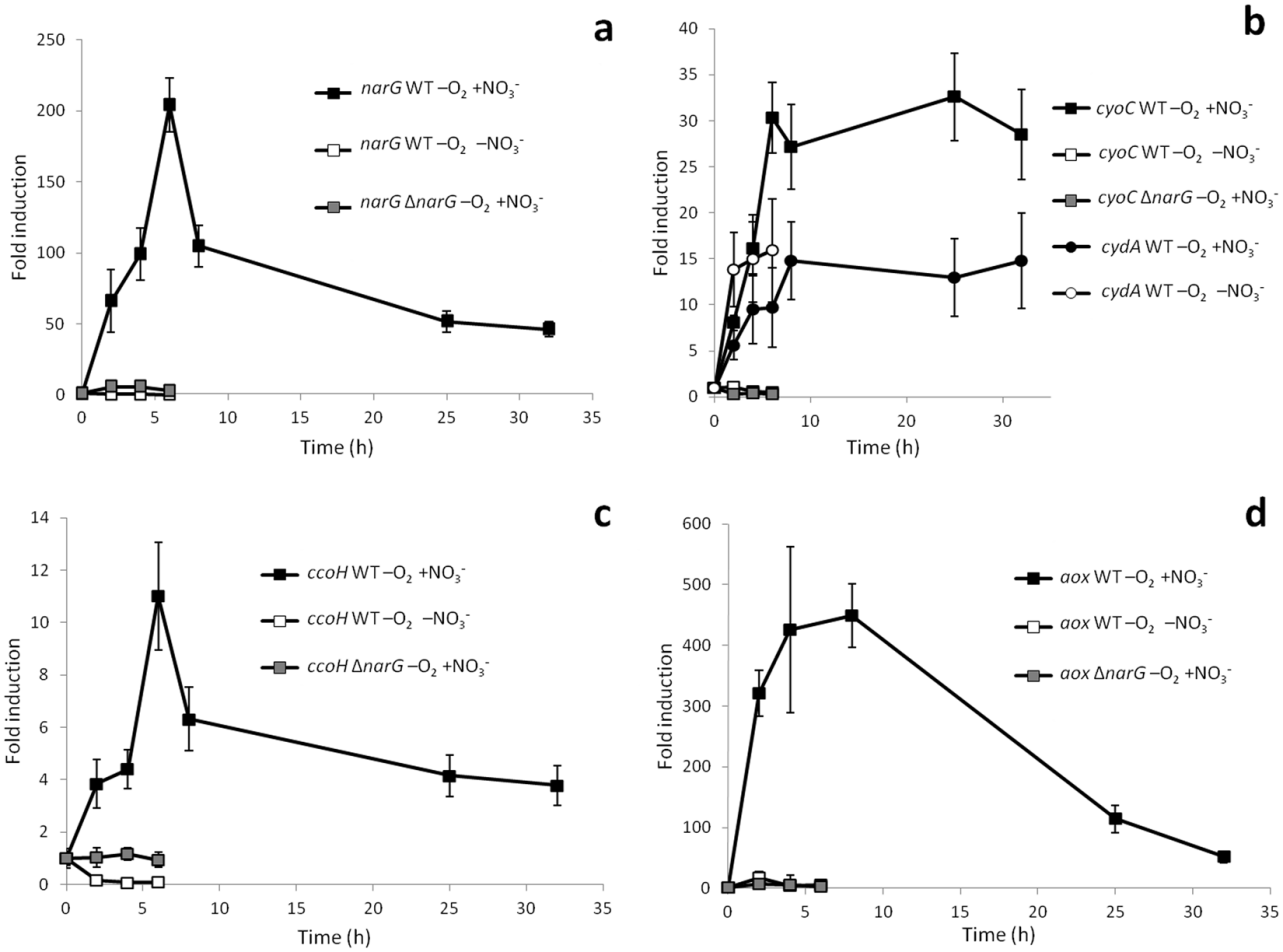
Induction kinetics of genes coding for electron transfer chains components. Anaerobic conditions with 20 mM nitrate in the WT strain are represented by black symbols, anaerobic conditions without nitrate in the WT strain by white symbols and anaerobic conditions with 20 mM nitrate in the Δ*narG* mutant by grey symbols. Fold induction of: (**a**) *narG* gene; (**b**) *cyoC* (squares) and *cydA* (circles) genes; (**c**) *ccoH* gene and (**d**) *aox* gene. As a control, expression kinetics of each gene in aerobiosis was performed along the growth curve and expression changed less than 3-fold. Fold change induction of each gene over time with respect to time 0 is shown and graphics represent the mean ± SD of 3–4 technical replicates.

Induction kinetics of *narG*, coding for the α subunit of the nitrate reductase, showed that this operon was induced up to 200-fold very fast (Fig. 2a), mostly within the first 6 hours, its transcription actually decaying at late exponential phase, when samples for dRNA-Seq were taken. In the absence of nitrate, expression of the *narG* gene was even repressed 10-fold when the culture was transferred to anaerobiosis (Fig. 2a). In order to analyse the requirement of nitrate or its respiration for anaerobic induction of a number of genes, an internal in-frame 3624 bp *narG* deletion mutant was constructed by a double recombination event (see Methods). As expected, the mutant was not able to anaerobically grow using nitrate as the electron acceptor (Supplementary Fig. S1). Anaerobic incubation of the *narG* mutant with nitrate resulted in induction of the *nar* operon (Fig. 2a) though to a much lesser extent than in the wild type strain (just 6-fold induction). This suggests that although nitrate might also be required for nitrate reductase induction, as in many other bacteria^17^, appropriate anaerobic respiration under this condition is strongly required for full induction.

The quinol oxidase *bo*3 is encoded by the *cyoABCDsurF1regBA* operon where all genes are overlapped or very closely located, thus also suggesting translational coupling among most of them. This operon was also clearly induced by anaerobiosis although to a lower extent than the *nar* operon (Table 1). It is intriguing that homologous genes in *E. coli* are regulated in an opposite way^18^. Induction kinetics of *cyoC* (Fig. 2b) showed a relatively fast induction of 30-fold within the first 6 hours, which was maintained along the whole exponential growth phase. As shown in Fig. 2b, anaerobic incubation of the WT strain in the absence of nitrate or the Δ*narG* mutant with nitrate did not result in *cyoC* induction at all, actually resulted in similarly weak repression of 3-fold, which indicated that nitrate has to be reduced in order to induce expression of this alternative electron acceptor. Interestingly, this operon was also aerobically induced to some extent by the NO-releasing molecule DETA-NO (Table 1). However, addition of DETA-NO to anaerobic cultures in the absence of nitrate did not result in *cyoC* induction (Supplementary Table S2).

The *cydAB* and *cydBD* genes coding for the quinol oxidase *bd* are encoded in two divergent operons, together with other membrane proteins and unknown proteins. Both divergent operons are induced in anaerobiosis to some extent (Table 1). Induction kinetics of *cydA* was very similar to that of *cyoC*, although the expression levels are lower. However, in this case it did not require nitrate at all (Fig. 2b), thus suggesting that *cydA* induction just required oxygen deprivation.

The *cco* genes coding for the cytochrome *c* oxidase *cbb*_3_ are clustered in the operon *ccoNOQPGHIS* (SGRAN_2449 to 2456). All of them seem to be part of an operon because they are overlapped or very close. Regulation seems to be complex because only the distal part of the operon (*ccoHIS*) appears to be induced in anaerobiosis. This was due to the higher expression levels of the proximal genes (*ccoNOQPG*) while growing in aerobiosis as compared to the distal genes (Supplementary Table S1). Intriguingly, the genes of the proximal part appeared to be repressed in aerobiosis by NO (Table 1), consistently with the reported strong inhibition of the *cbb*3-type oxidase by nitric oxide^19^, and by other stresses such as poor growth on tetralin as the carbon source (Supplementary Table S1).

Induction kinetics of *ccoH* indicated that a 10-fold induction is produced during the first 6 hours though expression was reduced later (Fig. 2c). Its expression was not affected in the *narG* mutant upon transfer to anaerobiosis in the presence of nitrate. However, *cooH* expression was repressed 10-fold when the wild type strain was anaerobically incubated in the absence of nitrate (Fig. 2c), thus suggesting that *ccoH* induction might also require nitrate itself, like *narG*, although its respiration is essential for full induction of this alternative electron acceptor.

According to the dRNA-Seq data, the gene coding for the alternative oxidase Aox, reported as nitric oxide insensitive, was also induced by anaerobiosis about 5-fold (Table 1). Interestingly, this gene was also aerobically induced by DETA-NO. Induction kinetics showed that induction by anaerobiosis was actually huge (450-fold) and very fast, reaching almost maximal induction after 4 hours in anaerobiosis (Fig. 2d), although its expression was subsequently decaying along the growth after 10 hours. In the absence of nitrate or in the *narG* mutant, a weak *aox* induction was also observed but to a much lower extent (17-fold). However, addition of NO in anaerobiosis in the absence of nitrate, resulted in an induction of 70-fold within the first 2 hours (Supplementary Table S2), thus suggesting that *aox* is mainly regulated by NO.

### Protein turnover genes

**Figure 3:**
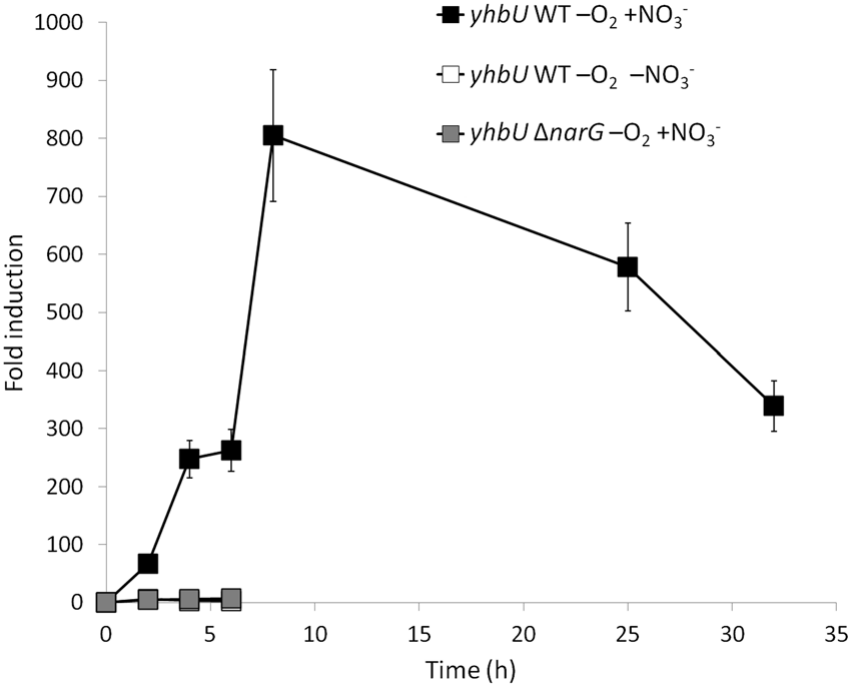
Induction kinetics of the *yhbU* gene, coding for a protease. Anaerobic conditions with 20 mM nitrate in the WT strain are represented by black squares, anaerobic conditions without nitrate in the WT strain by white squares and anaerobic conditions with nitrate 20 mM in the Δ*narG* mutant by grey squares. As a control, expression kinetics in aerobiosis along the growth curve was also performed and expression changed less than 2-fold. Fold change induction of the gene over time with respect to time 0 is shown and graphic represents the mean ± SD of 3–4 technical replicates.

The highest induced gene was found to be SGRAN_3859, putatively coding for a protease YhbU, which was virtually silent in aerobiosis and induced 300-fold in anaerobiosis (Table 1). The contiguous gene SGRAN_3860 (*yhbV*), apparently in the same operon and also coding for a protease, was also induced almost 100-fold. Out of the 20 genes coding for proteases, including the ClpP and HslV systems, Yhb proteases are the only ones induced in anaerobiosis. Induction kinetics of *yhbU* (Fig. 3) indicated that aerobic expression of this gene was always very low along the growth curve (not shown) but showed a strong induction of up to 800-fold within the first 8 hours in anaerobiosis. In anaerobiosis in the absence of nitrate or in the *narG* mutant in the presence of nitrate, *yhbU* showed a very weak induction of 6-fold within the first 2–4 hours (Fig. 3), thus suggesting that its induction requires nitrate respiration and probably growth under anaerobic conditions.

### Stress response/detoxification

A large number of genes presumably involved in response to stress conditions have been found induced in anaerobiosis. They are assigned to different COG classes but are included within the same physiological function in Table 1. Three operons coding for sulfide-quinone reductases, sulfurtransferases (some of them rhodanases) and transport proteins, presumably involved in sulfur detoxification, are anaerobically induced, which is consistent with an increase of SH_2_ under anaerobic conditions. Of these, the operon SGRAN_3840-3842 is also aerobically induced to some extent by DETA-NO.

Among these stress response operons, it is worth mentioning that the *ectABCD* operon presumably involved in biosynthesis of the ectoin osmoprotectant, which plays a role in osmotic stress response, is also induced in anaerobiosis. Similarly, genes coding for universal stress proteins and for an extracytoplasmic function sigma factor putatively involved in a general stress response (SGRAN_1161), were also induced in anaerobiosis but not by DETA-NO. Surprisingly, the stress response genes specifically induced by anaerobiosis also include a few coding for peroxidases, peroxiredoxins and a thioredoxin, which usually respond to oxidative stress.

Other operons presumably coding for stress response/detoxification proteins are not only induced by anaerobiosis but also aerobically induced by DETA-NO even to higher levels. These include the arsenic resistance operon *arsR3H2C3B2*, and the *norB* gene coding for an extended nitric oxide reductase that contains both subunits the of the NO reductase joined together. The most highly induced operon by either anaerobiosis or DETA-NO (100 to 200-fold, see Table 1) was SGRAN_3394 to SGRAN_3396, which apparently codes for proteins involved in traffic of proteins across the membrane, although their function could not be well defined from their sequence homology. Interestingly, the divergent *nsrR* gene (SGRAN_3393), putatively coding for a repressor sensing nitric oxide *via* a 2Fe-2S cluster^20^, was also induced by anaerobiosis or DETA-NO to some extent. Similarly high induction by either anaerobiosis or DETA-NO was also observed in *ytfE*, coding for the Iron-sulfur cluster repair protein YtfE. Induction under the same conditions although to a much lower extent was also observed in the operon SGRAN_1371 to SGRAN_1365, consisting of a transcriptional regulator gene followed by the *sufBCDS* genes, coding for the components of the Fe-S cluster biogenesis apparatus, and two genes coding for Fe-S assembly proteins.

**Figure 4:**
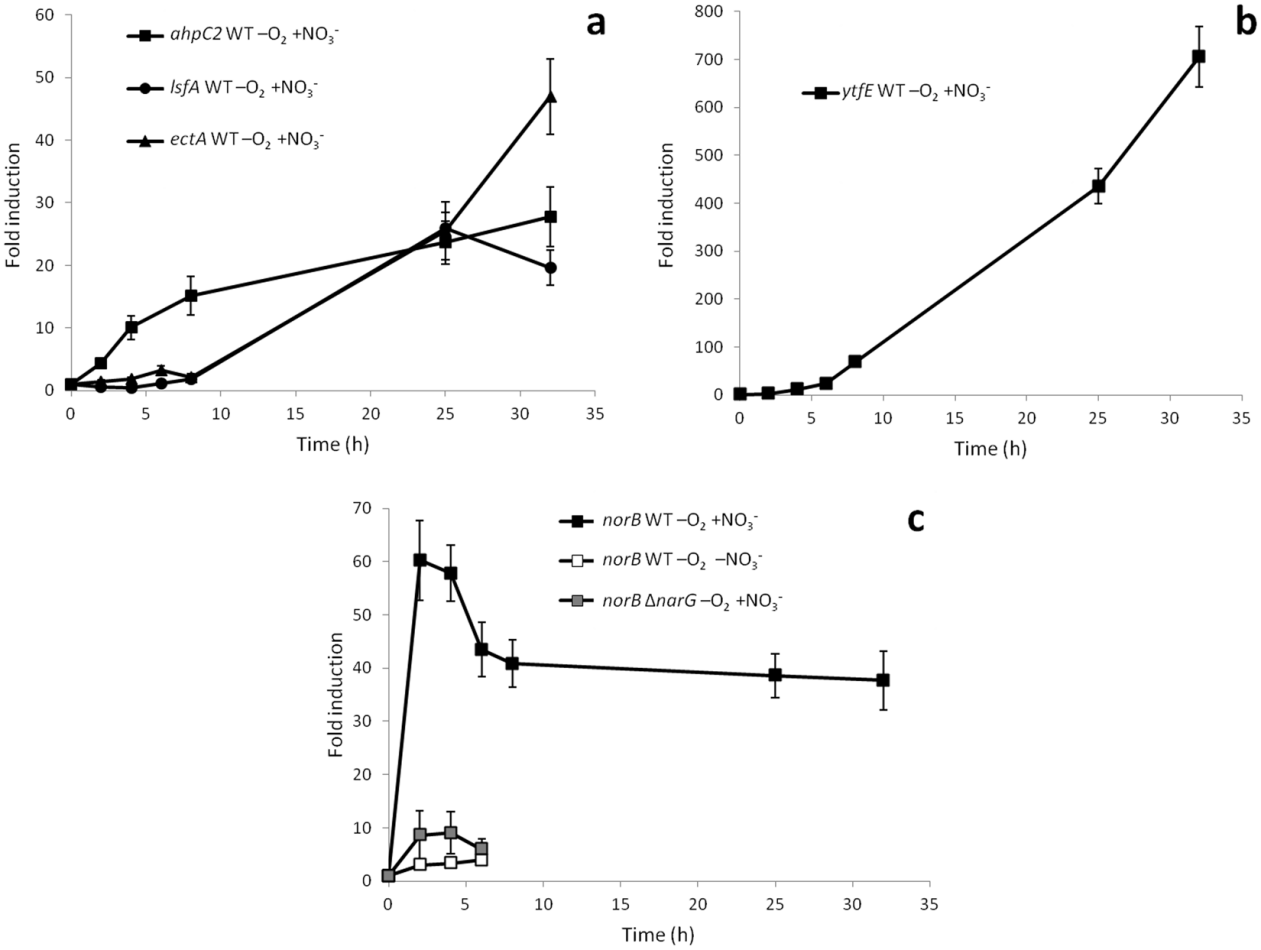
Induction kinetics of genes involved in stress response and detoxification. Anaerobic conditions with 20 mM in the WT strain are represented by black symbols, anaerobic conditions without nitrate in the WT strain by white symbols and anaerobic conditions with nitrate 20 mM in the Δ*narG* mutant by grey symbols. (**a**) Induction of *ahpC2* (squares), *lsfA* (circles) and *ectA* (triangles) genes. (**b**) Induction of *ytfE*. (**c**) Induction of *norB*. As a control, expression kinetics of each gene in aerobiosis was performed along the growth curve and expression changed less than 4.5-fold. Fold change induction of each gene over time with respect to time 0 is shown and graphics represent the mean ± SD of 3–4 technical replicates.

Induction kinetics in anaerobiosis has been analysed for the stress response genes *ahpC2, lsfA* and *ectA*, and for the *norB* and *ytfE* genes, which also were aerobically induced by DETA-NO. As shown in Fig. 4a, induction of *ahpC2* was relatively fast although it took more than 10 hours to reach its maximum. Induction of the other stress response genes was even slower, which suggested that some form of stress to which these genes respond is being progressively accumulated during anaerobic growth. Similarly, slow induction kinetic was observed for *ytfE* (Fig. 4b), which suggests that during growth in anaerobiosis the Fe-S clusters may have lost the iron and need to be repaired or *de novo* synthesized, thus explaining progressive induction of the required genes.

On the other hand, induction of *norB*, coding for the detoxifying nitric oxide reductase was very fast, reaching a maximum (60-fold) within the first 2 hours, which indicated that this gene is responding to a different condition (Fig. 4c). In the absence of nitrate or in the *narG* mutant, induction was similarly very weak (4 to 8-fold), which suggested that its induction requires nitrate respiration. Addition of DETA-NO to anaerobic cultures without nitrate resulted in a strong induction of both *norB* (24-fold) and *ytfE* (327-fold) within the first 2 hours (Supplementary Table S2). However, their different induction kinetics in anaerobiosis suggests that *norB* directly responds to NO whilst *ytfE* responds to the nitrosative damage generated by accumulation of NO.

### Replication, cell cycle control and cell division genes

**Figure 5:**
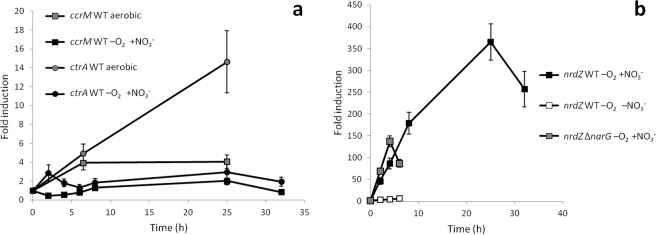
Induction kinetics of genes involved in replication, cell cycle control and cell division. (**a**) Fold induction of *ccrM* (squares) and *ctrA* (circles) genes. Anaerobic conditions with 20 mM nitrate in the WT strain are represented by black symbols and aerobic conditions in the WT strain by grey symbols. (**b**) Fold induction of *nrdZ* gene. Anaerobic conditions with 20 mM nitrate in the WT strain are represented by black squares, anaerobic conditions without nitrate in the WT strain by white squares and anaerobic conditions with 20 mM nitrate in the Δ*narG* mutant by grey squares. As a control, expression kinetics of *nrdZ* in aerobiosis was performed along the growth curve and expression changed less than 2.5-fold. Fold change induction of each gene over time with respect to time 0 is shown and graphics represent the mean ± SD of 3–4 technical replicates.

The genes coding for the known cell cycle regulators in α–proteobacteria CtrA and CcrM^21,22^ were found repressed by 3.5- and 7-fold, respectively. A gene coding for a chromosome partitioning protein (SGRAN_3445) or the *ftsN, ftsB* and *ftsW* genes involved in cell division, were similarly repressed by 3- to 6-fold (Table 1), although other *fts* genes were not differentially regulated. However, unlike the gene expression machinery genes (e. g. SGRAN_2978), expression levels were not reduced for *ctrA* and only slightly reduced for *ccrM* under other slow growth condition in aerobiosis (Supplementary Table S1), thus suggesting that its repression in anaerobiosis was not just due to slow growth. Kinetics of the transcriptional response of *ccrM* and *ctrA* (Fig. 5a) showed differential expression of these genes in aerobiosis depending on the growth phase, transcription being progressively increased up to 15-fold along the growth curve in the case of *ctrA*. However, *ctrA* was induced in anaerobiosis less than 3-fold along the growth curve, and *ccrM* showed a fast transcriptional repression within the first two hours. Nevertheless, *ccrM* expression was recovered after 6 hours, although its level was lower than that when growing in aerobiosis, until late exponential phase, when its expression decayed again. This is consistent with the lag phase observed when the culture was transferred to anaerobic conditions. It appears as if TFA cells responded to anaerobiosis by arresting cell division upon transferring them to the new condition, probably until the global transcriptional profile is rearranged and the machinery required for growth under anaerobic nitrate respiration conditions produced.

In agreement with a slower growth rate, a few genes required for NTPs biosynthesis (COG code F) such as the gene coding for the nucleoside diphosphate kinase, (SGRAN_2978), were also repressed (Table 1). On the contrary, *nrdZ*, which codes for an O_2_-independent ribonucleotide reductase essential for DNA synthesis under anaerobic conditions^23^ was progressively induced up to 365-fold. Anaerobic incubation in the absence of nitrate resulted in induction to a much lower extent (6-fold). However, this gene was initially induced in the *narG* mutant incubated in the presence of nitrate, although induction subsequently decayed, thus suggesting that its induction did require nitrate though maximal expression required respiration (Fig. 5b).

### SOS DNA repair system

A striking result from the global response to anaerobic growth is that many genes typically involved in the SOS response to DNA damage in other bacteria are induced during anaerobic growth. These include the *lexA* repressor, *recA*, whose product controls LexA activity by proteolysis in response to DNA damage, the two operons coding for error-prone DNA polymerases able to carry out DNA translesion synthesis, and genes of uncertain function but coding for proteins containing a SRAP domain, which functions as a DNA-associated autoproteolytic switch that recruits diverse repair enzymes upon DNA damage^24^.

**Figure 6:**
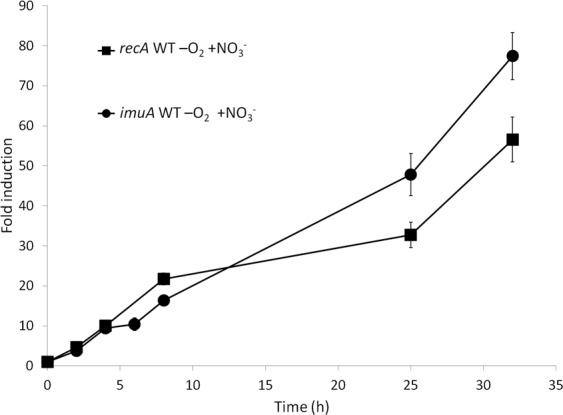
Induction kinetics of genes of the SOS DNA repair system. Induction of *recA* (squares) and *imuA* (circles) in anaerobic conditions with 20 mM nitrate in the WT strain. As a control, expression kinetics of each gene in aerobiosis was performed along the growth curve and expression changed less than 3-fold. Fold change induction of each gene over time with respect to time 0 is shown and graphic represents the mean ± SD of 3–4 technical replicates.

Induction kinetics of *recA* and *imuA* (Fig. 6) clearly showed a slow induction, thus suggesting that the signal to which their expression responds, presumably DNA damage, need to be accumulated during growth in anaerobiosis.

### Bacterial appendages, motility and adherence

In strain TFA there are 39 contiguous putative flagellar genes (SGRAN_4088 to SGRAN_4126), apparently arranged in at least 4 operons, which include a putative sigma-54 dependent activator, the sigma-28 coding gene *fliA* and its anti-sigma *flgM*, and two flagellin-coding genes, *fliC* and *fliC2*. All genes were repressed during growth in anaerobiosis (for simplicity, only a few flagellar genes representative of the 4 operons are shown in Table 1; for differential expression of other flagellar genes, see Supplementary Table S1). Similarly, 6 out of the 8 putative chemotaxis genes annotated in the genome were repressed at least 5-fold (Table 1).

**Figure 7:**
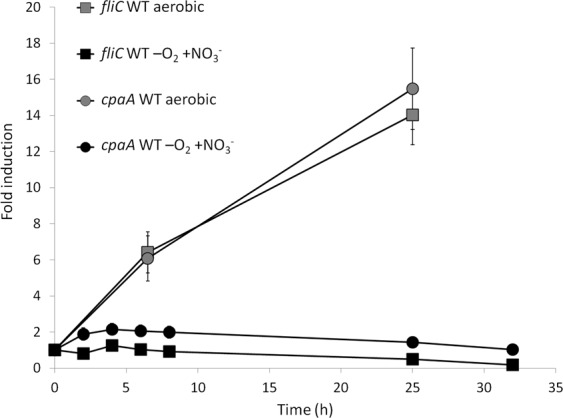
Regulation kinetics of flagellar and pili genes in TFA. Regulation of *fliC* (squares) and *cpaA* (circles) genes. Aerobic conditions in the WT strain are represented by grey symbols and anaerobic conditions with 20 mM nitrate in the WT strain by black symbols. Fold change induction of each gene over time with respect to time 0 is shown and graphic represents the mean ± SD of 3–4 technical replicates.

Regulation kinetics of *fliC* and *fliC2* genes (Fig. 7), indicated that flagellar gene expression increases with the growth phase while growing in aerobiosis. However, the *fliC* genes were not induced along the growth curve in anaerobiosis.

**Figure 8:**
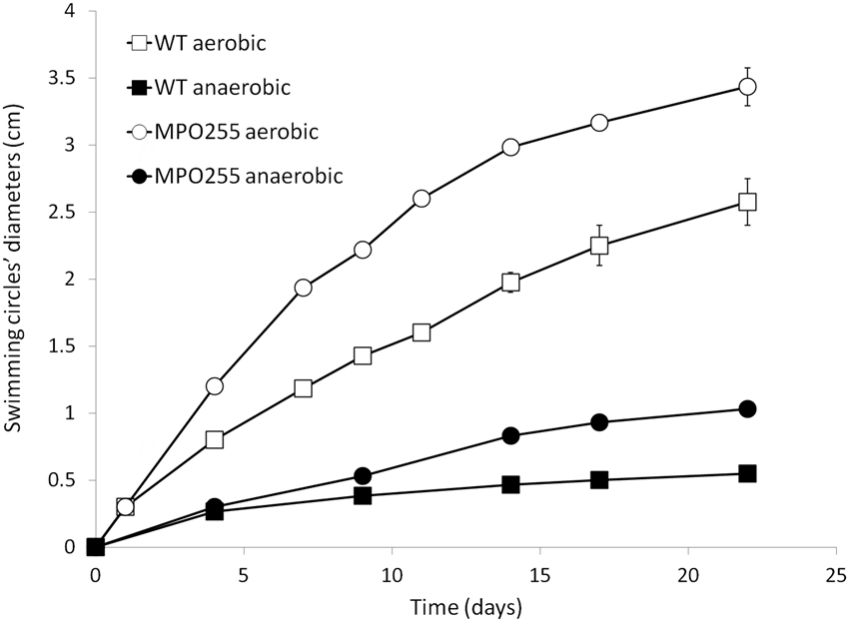
Swimming motility assays. Graphs represent the evolution of the swimming circles diameter of WT strain (squares) and the spontaneous swimming mutant MPO255 (circles) in aerobic (white) and anaerobic (black) conditions. Graphic represents the mean ± SD of 3–4 biological replicates.

Repression of the flagellar and chemotaxis genes in anaerobiosis suggested that motility could be severely reduced under this condition. To empirically test this, swimming motility assays were performed in aerobiosis and in anaerobiosis. As shown in Fig. 8, the strain TFA was barely able to swim while growing in anaerobiosis. During these assays, a spontaneous mutant was isolated that showed increased motility in aerobiosis. Motility of this mutant was also tested in anaerobiosis and showed higher motility than the wild type strain, thus indicating that the extremely reduced motility shown by the wild type strain in anaerobiosis is not simply a consequence of energy limitation.

In addition to the flagellar genes, the strain TFA also codes for a type IVb pili. All the 13 genes involved in pilin secretion and Flp pilus assembly (*cpa*, *tad, pilZ*) were also repressed when growing under anaerobic conditions. Induction kinetics of *cpaA* showed that it coregulated with the flagellar genes (Fig. 7).

## Discussion

Differential RNA-Seq of *S. granuli* strain TFA while growing under aerobic or anaerobic conditions has revealed an extensive change in the global transcriptomic profile that probably has as a consequence a strong difference in the proteome composition in both conditions. Among the most hugely induced genes are those coding for the proteases YhbV and HybU. Although to a lesser extent, these or other proteases were also anaerobically induced in *E. coli*^25^, *Shigella flexneri*^26^*, Yersinia intermedia*^27^*, Pseudomonas aeruginosa*^28^ and *Corynebacterium glutamicum*^29^. This suggests that in order to achieve a large change in the proteome profile, above all in an energy limiting condition such as the transition to an anaerobic environment, it may be advantageous to actively degrade proteins not required under the new situation to facilitate a fast reprograming of the bacterial protein pattern in order to optimize its adaptation to the new environmental condition. A similar situation was also observed in *P. putida*, in which genes coding for components of a putative proteasome were among the most strongly induced genes during its adaptation to a nitrogen limiting conditions, which also involved extensive changes in genes expression^30^.

One obvious and expectable change implies induction of the alternative respiratory electron transfer chain that allows respiration by using nitrate as the final electron acceptor. However, anaerobiosis also resulted in a fast induction of other four terminal oxidases that transfer electrons to oxygen and, therefore, are useless for continuous growth under anaerobic conditions. One simple explanation, which might be valid for *cyd* genes, is that gene induction just responds to oxygen limitation, as it has been shown for high affinity terminal oxidases^17^. However, these oxidases are not actually induced under complete absence of oxygen in other bacteria^18^. Besides, active nitrate respiration is required for induction of the other 3 oxidases in the strain TFA. Induction of the alternative oxidase *aox* apparently responds to the presence of nitric oxide, which is consistent with its reported biochemical insensitivity to NO, its transcriptional activation by the nitric oxide sensor NrsR in other bacteria^31^ and its role in reducing stress in bacteria exposed to nitric oxide^32^. Regulation of the alternative terminal oxidases is complex and their functional role not completely characterised yet. For instance, it has been recently reported that the cytochrome *bd* oxidases of *E. coli* are essential for respiration under high H_2_S concentrations^33^. Anyways, in strain TFA we have observed 3 different induction profiles for these alternative oxidases. Induction under anaerobic conditions of these oxidases in the strain TFA may reflect a response to different particular signals, which might have functional relevance or, on the contrary, be gratuitous for its growth under anaerobic conditions.

Induction of a large number of different genes putatively involved in stress response or detoxification suggests that anaerobiosis is a hostile environment for strain TFA. Induction of some of these genes might just respond to anaerobiosis but many of them are not induced if the strain cannot use the alternative electron acceptor, thus suggesting that they may respond to some forms of stress inherent to nitrate respiration by this bacterium. Different responses of some of these genes suggest that there may be different stress signals.

One clearly stressing condition is accumulation of nitrite, the anaerobic respiration product in TFA, or its protonated form HNO_2_, which is toxic to many bacteria^34^ and the responsible for stopping anaerobic growth of the strain TFA^4^.

Another stress clearly suggested by induction of Fe-S cluster repair and biogenesis genes is that the iron sulphur clusters of proteins are significantly damaged during anaerobic growth. This is typical of nitrosative damage, primarily caused by accumulation of NO, which is consistent with the induction of these genes by NO. That anaerobic growth of strain TFA results in NO accumulation and nitrosative stress is also suggested by anaerobic induction of an NO-reductase (*norB*) and other genes that were also induced by NO both aerobically (Table 1) and anaerobically (Suplementary Table S2). Nitrosative stress and the response to it under anaerobic conditions are also well documented although knowledge on the precise mechanisms to sense this stress and cope with it is still incomplete^35,36^. Although NO accumulation in cultures of TFA growing anaerobically might result intriguing because it naturally lacks nitrite reductase, nitrite accumulation in sub-stoichiometric amounts in relation to the nitrate reduced has been systematically observed during anaerobic grow of strain TFA (Supplementary Fig. S1 and^4^), which suggest that 4–5 mM of nitrate reduced to nitrite is further metabolised to other nitrogen forms, presumably to NO, and, subsequently, to N_2_O by the action of the NO-reductase NorB. Which enzyme might be producing NO is unknown but there is extensive evidence clearly indicating that the enteric nitrate reductase NarG plays a major role in causing nitrosative stress during anaerobic nitrate respiration by producing most of the NO^35^.

Particularly intriguing is the induction of genes normally involved in oxidative stress. Actually, a number of homologous genes, including those involved in Fe-S cluster biogenesis are induced in *E. coli* when oxygen is supplied to anaerobic cultures^37^. However, the catalase KatA is required for anaerobic growth in *P. aeruginosa*^38^. Also in *N. gonorrhoeae*, many genes found to be responsive to anaerobiosis have also been shown to be responsive to iron and/or oxidative stress^39^.

Adaptation to stressing conditions under anaerobiosis is apparently also extended to the ribosomes since *rlmH* is induced. RlmH methylates the pseudouridine 1915 of the 23 S rRNA that apparently provides fitness advantage under stress conditions^40^, an additional argument supporting that anaerobiosis is a hostile environment to TFA. The homologous gene is not induced under anaerobiosis in *E. coli*^37^ or *Shigella*^26^, but induced by other forms of stress, which suggests that growth under anaerobic conditions is particularly stressing for this strain.

Anaerobic growth by respiring nitrate may not be just stressing but also mutagenic to strain TFA, since genes of the SOS DNA repair system, including the repressor *lexA*, *recA*, and two operons coding for error-prone DNA polymerases, were also induced during growth under these conditions. This SOS response while growing anaerobically is unusual and only reported in *Corynebacterium glutamicum*^29^, which only reduced nitrate while accumulating nitrite, as strain TFA does, and *Neisseria gonorrhoeae*^39^, which was grown on nitrite as the electron acceptor. Nitrous acid has been reported to be mutagenic although it actually is dinitrogen trioxide the mutagenic agent^41^. Dinitrogen trioxide can be produced from NO·, although it requires oxygen, or by condensation of two molecules of nitrous acid under anaerobic conditions^41^. Although production N_2_O_3_ from NO_2_H at neutral pH is low because of its low pKa (3.16 at 25 °C), these growth conditions in the presence of high concentrations of nitrite may produce sufficient N_2_O_3_ to induce DNA lesions and the subsequent SOS response.

Finally, a striking phenomenon observed during anaerobic growth is the lack of expression of genes involved in production of the bacterial appendages or in chemotaxis, which resulted in dramatic reduction of the motility capacity of the bacterium. In enterobacteria^42^ and other bacteria^43^, flagellar biosynthesis is not reduced but even increased in anaerobic condition, and is regulated by ArcA to facilitate its motility in search for nutrients^44^. However, a weak repression of some flagellar genes in anaerobiosis has also been reported in other bacteria^28,45^. Also, repression of flagellar genes has been reported when cells of *Sphingobium* are exposed to high concentrations of nickel^9^, thus suggesting that it might be part of a response to stressing conditions.

Expression of flagellar and pili genes in strain TFA is dependent of the growth phase (Fig. 7), as the cell cycle regulator genes (Fig. 5), and flagellum production has been reported to be cell cycle-dependent and activated by CtrA in *Caulobacter crescentus*^46^ and other α-proteobacteria^47,48^, including the closely related *Sphingomonas melonis*^49^. Thus, lack of flagellar gene expression in strain TFA while growing in anaerobiosis could be explained by similar lack of expression of *ctrA*.

## Methods

### Bacterial strains, plasmids and primers

*S. granuli* strains used in this study were wild type *S. granuli* strain TFA, a mutant derivative with an almost complete deletion of *narG* gene, MPO253, and a spontaneous mutant with increased motility, MPO255. *Escherichia coli* strain DH5α^50^ and DH5α λpir were also used for cloning. Previously described plasmids used were pSW-I^51^, pAK405^52^ and pEMG^53^. Primers used in this work are listed in Supplementary Table S3.

For the construction of pMPO1412, a fragment containing *rpsL1* gene conferring sensitivity to streptomycin to naturally occurring streptomycin-resistant bacteria, was amplified from pAK405 using primers rpsL1 fw and rpsL1 rv (Supplementary Table S3), and cloned in pEMG vector using the restriction enzyme AflIII from New England Biolabs (ends blunted with Klenow from New England Biolabs). The construction was checked by sequencing.

The *S. granuli* TFA deletion mutant in *narG* gene was constructed using a variation described below of a previously described method^53^. TFA cells were grown in MML rich medium^11^ at 30 °C both in liquid culture and plates. Antibiotic concentrations used for TFA were streptomycin 50 or 200 μg/mL, kanamycin 20 μg/mL and ampicillin 5 μg/mL. Regions 1 kb upstream and downstream of the *narG* were amplified by PCR with the primers narG F1F, narG F1R, narG F2F and narG F2R (Supplementary Table S3) and were cloned in pMPO1412 vector using the restriction enzymes SacI and BamHI for the upstream region and BamHI and XbaI for the downstream region (New England Biolabs). The construction was checked by sequencing. The generated plasmid containing the 3624 bp deletion of *narG* was transformed by electroporation in *S. granuli* TFA wild type strain. Cells resistant to Kanamycin but sensitive to Streptomycin (200 mg/L), which should have the plasmid integrated in the chromosome by homologous recombination, were selected and the candidate cells checked by PCR with the primers rpsl1 fw and rpsl1 rv (Supplementary Table S3). A positive candidate was electroporated with pSW-I plasmid and candidates resistant to ampicillin and streptomycin (50 mg/L) resistant but sensitive to kanamycin, which should have undergone the second recombination event, were selected. Candidates were checked by PCR with the primers F1F and narG qPCR R (Supplementary Table S3) and by anaerobic growth incapability phenotype. Positive candidates were grown for several generations without ampicillin and ampicillin sensitive candidates that have lost the pSW-I plasmid were selected.

### Sample preparation for high-throughput RNA sequencing

For RNA-Seq analysis we used RNA from TFA WT cells in different growth conditions, namely: (i) anoxic condition with nitrate using BHB as the carbon and energy source, “Anaerobiosis” in Supplementary Table S1; (ii) aerobiosis using tetralin as the carbon and energy source, “Tetralin” in Supplementary Table S1; (iii) aerobiosis using BHB as the carbon and energy source in the presence of nitric oxide, “DETA-NO” in Supplementary Table S1; and (iv) aerobic growth using BHB as the carbon and energy source, “Aerobiosis” in Supplementary Table S1. For detailed description of the methods used for sample preparation and bioinformatic analysis of the data see Supplementary Information.

### RT-qPCRs assays

To validate the RNA-Seq analysis and to study the expression levels of selected genes along the growth curves, we performed experiments with cultures grown under several conditions. For those conditions in which cells were unable to grow, we took as initial OD_600_ the same used for the RNA-seq (see Supplementary Information) and took samples at shorter times, while for those conditions in which cells were able to grow we performed a complete growth curve from low OD_600_ to see the evolution of the expression of genes through the growth curve. The chosen conditions were the following: (i) For the wild type strain in aerobic conditions, and in anaerobic conditions with 20 mM of nitrate, cells were grown aerobically in minimal medium until the exponential phase and then diluted to an initial optical density of about 0.1 in the same media. After one hour of aerobic incubation, the initial time 0 was taken, the culture was split, sodium nitrate was added to the one half of the culture and then incubated under anaerobiosis during the rest of the assay. The other half was incubated under aerobic conditions. Three independent biological replicates of each growth condition were treated for RNA purification as explained below. (ii) For the wild type in anaerobic conditions without nitrate, or without nitrate but DETA-NO and Δ*narG* mutant in anaerobic conditions with nitrate, cells were grown in minimal medium in aerobic conditions until an optical density of about 0.7. Then, time 0 was taken and the cultures were incubated under anaerobic conditions either in the absence of nitrate or with 4 mM DETA-NO for the wild type, or with nitrate for the Δ*narG* mutant. Three independent biological replicates of each growth condition were treated for RNA purification as explained below.

Total RNA extraction was carried out as previously described^54^. DNase I treatment was performed with a DNA-free kit (Ambion). The samples were purified using RNAeasy columns (Quiagen) and RNA quality was confirmed by non-denaturing agarose gel electrophoresis. The absence of contaminating DNA was then confirmed by PCR amplification. The three replicates of each sample were mixed so that the same RNA quantity of each replicate was added to the mix. Retro-transcription of these RNA mixes was performed using the High-Capacity cDNA Archive Kit (Applied Biosystems), with random hexamers as primers to generate cDNAs. The resulting cDNA samples were amplified by RT-qPCR using 0.3 mM of each primer (Supplementary Table S3) as previously described^55^. The results are the average of 3-4 technical replicas. In all conditions fold change induction with respect to time 0 is represented.

### Swimming assays and swimming mutant isolation

MML rich medium plates and minimal medium plates with 40 mM BHB^11^ and streptomycin with 0.3% agar were prepared and left for 4 hours at room temperature to polymerize. One colony of WT TFA was punctured in each plate inside the agar and plates were incubated aerobically upside up at 30 °C.

During these assays, “bubbles” of faster swimming biomass were observed in some of the swimming circles. Biomass from these bubbles was taken and used to run new swimming assays in minimal medium and in rich medium. Biomass from the external part of the larger swimming halos was streaked in MML plates that were incubated at 30 °C for 4 days to isolate spontaneous mutants with increased motility.

For swimming assays, colonies of the WT TFA strain and the spontaneous swimming mutant MPO255 were punctured in minimal medium swimming plates without nitrate for aerobic conditions and with nitrate 20 mM for anaerobic conditions. Plates were incubated at 30 °C, in anaerobic jars in the case of anaerobic conditions where anaerobiosis was reached by the use of Anaerocult A reactive (Merk) and checked by Microbiology Anaerotest indicator (Merck). Three biological replicates of each strain were carried in each condition. Plates were incubated at 30 °C and the diameters of the swimming circles generated at different days were measured.

## Supplementary information

Supplementary information

Supplementary dataset 1

## Data Availability

All data generated or analysed in this study are included in this article.
