# Supplementary material for: The response of Sphingopyxis granuli strain TFA to the hostile anoxic condition
Source: Sci Rep. 2019 Apr 18;9:6297. doi: 10.1038/s41598-019-42768-9 (PMC6472365; doi:10.1038/s41598-019-42768-9)
Supplement: Supplementary file 1 — Supplementary information [file 41598_2019_42768_MOESM1_ESM.pdf]

SUPPLEMENTARY INFORMATION

The response of *Sphingopyxis granuli* strain TFA to the hostile anoxic condition

Yolanda Elisabet González-Flores, Ruben de Dios, Francisca Reyes-Ramírez* and Eduardo Santero.

Sample preparation for high-throughput RNA sequencing:

For all growth conditions, TFA was previously grown aerobically at 30°C in mineral medium containing 40 mM BHB as the only carbon and energy source to the exponential phase (optical density at 600 nm, 0.8). Then, cells were diluted into a fresh medium to an initial optical density of about 0.1. All cultures were grown at 30°C until they reached a final optical density of 0.7, when samples for RNA extraction were taken. Aerobic conditions are represented as “Aerobiosis” in Supplementary Table S1. In the case of the anaerobic cultures (“Anaerobiosis” in Supplementary Table S1), the appropriate aliquots were transferred into standing stoppered bottles filled to the top and with sodium nitrate 20 mM as a final electron acceptor [1]. Anaerobic growth of TFA was routinely followed together with nitrate consumption and nitrite accumulation [1,2]. For the cultures in the presence of DETA-NO (“DETA-NO” in Supplementary Table S1), 4 mM of the NO releaser was added to the aerobic cultures and incubated just for one additional hour at 30 ºC to prevent differences in cell density with respect the standard aerobic culture due to NO toxicity. For tetralin-grown cells (“Tetralin” in Supplementary Table S1), tetralin was supplied as a sole carbon and energy source in the gas phase as previously described [3]. The concentration of antibiotic used for TFA was streptomycin 50 μg/ml

Total RNA extraction was carried out as previously described [4] from exponentially-growing TFA cells in each of the different growth conditions. DNase I treatment was performed with a DNA-free kit (Ambion). The samples were purified using RNAeasy columns (Quiagen) and RNA quality was confirmed by non-denaturing agarose gel electrophoresis. The absence of contaminating DNA was then confirmed by PCR amplification. Equal

amounts of RNA obtained from three independent biological replicates of each growth condition were mixed and used in the gene expression analysis. RNA samples were sent to the ASCIDEA Headquarters, Barcelona (Spain) (http://www.ascidea.com/lifesciences-services.html) for library preparation and RNA sequencing. The cDNA libraries were sequenced using an Illumina HiSeq2000 machine.

Bioinformatic analysis

The bioinformatic analyses were carried out by ASCIDEA. Briefly, quality of the reads obtained by HiSeq2000 sequencing was checked with FastQC software (http:/www.bioinformatics.bbsrc.ac.uk/projects/fastqc/). Preprocessing of the reads was performed with fastx-toolkit (http://hannonlab.cshl.edu/fastx_toolkit/index.html) and aScidea specific perl scripts property of aScidea (http://www.ascidea.com) in order to filter regions of low quality. Adaptors were trimmed and low quality bases at the ends of sequences and reads with undetermined bases or with 80% of their bases with less than 20% quality score. Raw reads that passed the quality filter threshold were mapped using Bowtie2 2.0.6 [5]. The reference genome used and genomic annotations were obtained from NCBI public databases (https://www.ncbi.nlm.nih.gov/nuccore/CP012199.1) [1]. The inner distance between mate pairs used was 50 bp and the rest of the parameters were used with the default values. Gene level counts were calculated and FPKM normalized using Cufflinks 2.0.2 software [6]. Differential transcript expression was then computed using Cuffdiff. Main statistical analyses were performed using the free statistical language R and the libraries developed for data analysis by the Bioconductor Project (www.bioconductor.org) [7]. FPKM values were used to normalise and quantify the gene expression level. Genes differentially expressed more than 3-fold between the different conditions studied were selected for further analysis. These genes were clustered according to the Cluster of Orthologous Groups of proteins (COGs) broad classification [8].

Supplementary Table S2. Gene induction after 2 hours of addition of DETA-NO. Fold induction was calculated with respect to time 0 before addition of DETA-NO.

Gene	Fold induction
ytfE	327 ± 64
aox	74 ± 16
norB	24 ± 5
nsrR	7 ± 1
narG	2.4 ± 0.5
ccoH	0.7 ± 0.1
cyoC	0.3 ± 0.3

Supplementary Table S3. Primers used in this work.

Name	Sequence 5' --> 3'	Function
narG qPCR F2	GCTGACCTGGTTCGTCTAC	Forward primer for *narG* gene RT-qPCR
narG qPCR R2	AGCTGGCTCATGCGGAAAC	Reverse primer for *narG* gene RT-qPCR
cyoC qPCR F	GAGCCGGTGTCCTTCTATC	Forward primer for *cyoC* gene RT-qPCR
cyoC qPCR R	CCGCCGAGCACCGCATAG	Reverse primer for *cyoC* gene RT-qPCR
cydA qPCR F	TCCAGTTCGGCACCAACTG	Forward primer for *cydA* gene RT-qPCR
cydA qPCR R	GCTTGTCCCATCCGAAGAAC	Reverse primer for *cydA* gene RT-qPCR
ccoH qPCR F	GTTTCTTCGGCACGATCATC	Forward primer for *ccoH* gene RT-qPCR
ccoH qPCR R	CATCGTCGAGCCACTTGTTG	Reverse primer for *ccoH* gene RT-qPCR
aox qPCR F	CGCGGTCGTGCTCGAAAC	Forward primer for *aox* gene RT-qPCR
aox qPCR R	GTCTTGATCCAGCCCTTGTC	Reverse primer for *aox* gene RT-qPCR
yhbU qPCR F	GGCAGCCCGGAGCTCATC	Forward primer for *yhbU* gene RT-qPCR
yhbU qPCR R	TGAAGTTGAGGCCGGAGAAG	Reverse primer for *yhbU* gene RT-qPCR
ahpC2 qPCR F	TCCATGCCGCGGATCAAC	Forward primer for *ahpC2* gene RT-qPCR
ahpC2 qPCR R	GTGCAGACCGGCGTGAAG	Reverse primer for *ahpC2* gene RT-qPCR
lsfA qPCR F	CAGGCCGCACCCGATTTTG	Forward primer for *lsfA* gene RT-qPCR
lsfa qPCR R	GTGCAGATCGGCGTATAATC	Reverse primer for *lsfA* gene RT-qPCR
ectA qPCR F	GATGCCGGGTCTGCAACTG	Forward primer for *ectA* gene RT-qPCR
ectA qPCR R	ATGCACTGATCGGCGAAATG	Reverse primer for *ectA* gene RT-qPCR
ytfE qPCR F	GCAAGCAATCCCGTCGAAAC	Forward primer for *ytfE* gene RT-qPCR
ytfE qPCR R	GACGCGCTGTGCCATCTG	Reverse primer for *ytfE* gene RT-qPCR
norB qPCR F	GGGGATTTCCGGGGTAGAG	Forward primer for *norB* gene RT-qPCR
norB qPCR R	ATCGAGCCGAGCTGCATTC	Reverse primer for *norB* gene RT-qPCR
ccrM qPCR F	GCATCCTGCAGGGCGATTG	Forward primer for *ccrM* gene RT-qPCR
ccrM qPCR R	GAGGTCGCCGCCGAGTTG	Reverse primer for *ccrM* gene RT-qPCR
ctrA qPCR F	GACGACCGAGGGCTTCAAC	Forward primer for *ctrA* gene RT-qPCR
ctrA qPCR R	CAATTTCTTGAGCACGTCATAG	Reverse primer for *ctrA* gene RT-qPCR
nrdZ qPCR F	CTGCGAGCGGGAGTTTCTG	Forward primer for *nrdZ* gene RT-qPCR
nrdZ qPCR R	GTCGAGATCGTCGGGAATC	Reverse primer for *nrdZ* gene RT-qPCR
recA qPCR F	ACAATTGTCACTCGTCGAATC	Forward primer for *recA* gene RT-qPCR
recA qPCR R	CTTCGAGCCGAGCTTCATC	Reverse primer for *recA* gene RT-qPCR
imuA qPCR F2	GACGCGCTTCGATCTTTATG	Forward primer for *imuA* gene RT-qPCR
imuA qPCR R2	AGGTCGCGGTCGGATCTG	Reverse primer for *imuA* gene RT-qPCR
fliC qPCR F	ACTGTCATCAACACCAATGTG	Forward primer for *fliC* gene RT-qPCR
fliC qPCR R	GTCCTTCGCGCTGTTGATG	Reverse primer for *fliC* gene RT-qPCR
cpaA qPCR F	GATCTCGAACGGGCTGAAC	Forward primer for *cpaA* gene RT-qPCR
cpaA qPCR R	CGGCGAACAGCAGGAATAC	Reverse primer for *cpaA* gene RT-qPCR
rpsl1 Fw	GTTCGCTAGGGCCCATGG	Primer forward to amplify the streptomycin sensitivity gene of pAK405 and pMPO1412 plasmids
rpsl1 Rv	AATCCTGCTCTGCGAGGC	Primer reverse to amplify the streptomycin sensitivity gene of pAK405 and pMPO1412 plasmids
narG F1F	CAAAGCTTGGATCCGTCTTGCG	Forward primer for the amplification of the upstream region of *narG* gene in TFA
narG F1R	TCGGGGATCCTTTGCTTCGTCTTG	Reverse primer for the amplification of the upstream region of *narG* gene in TFA
narG F2F	AATATGGGATCCTCCGCAAATTG	Forward primer for the amplification of the downstream region of *narG* gene in TFA
narG F2R	GCAGGTTGTCTAGATATTTGAGC	Reverse primer for the amplification of the downstream region of *narG* gene in TFA
narG qPCR R	CAGGAGCCGGTGCAGTTC	Reverse primer used to check Δ*narG* mutant

Supplementary Figure S1. Anaerobic incubation with nitrate of WT strain and Δ*narG* mutant MPO253. WT strain is represented by black symbols and Δ*narG* mutant by white symbols. OD_{600} (squares) was measured for both strains over time and nitrate (circles) and nitrite (triangles) concentrations were measured for the WT strain. The time in which the samples for the RNA-seq were taken is indicated by the arrow. Graphic represents the mean ± SD of 3-4 biological replicates.

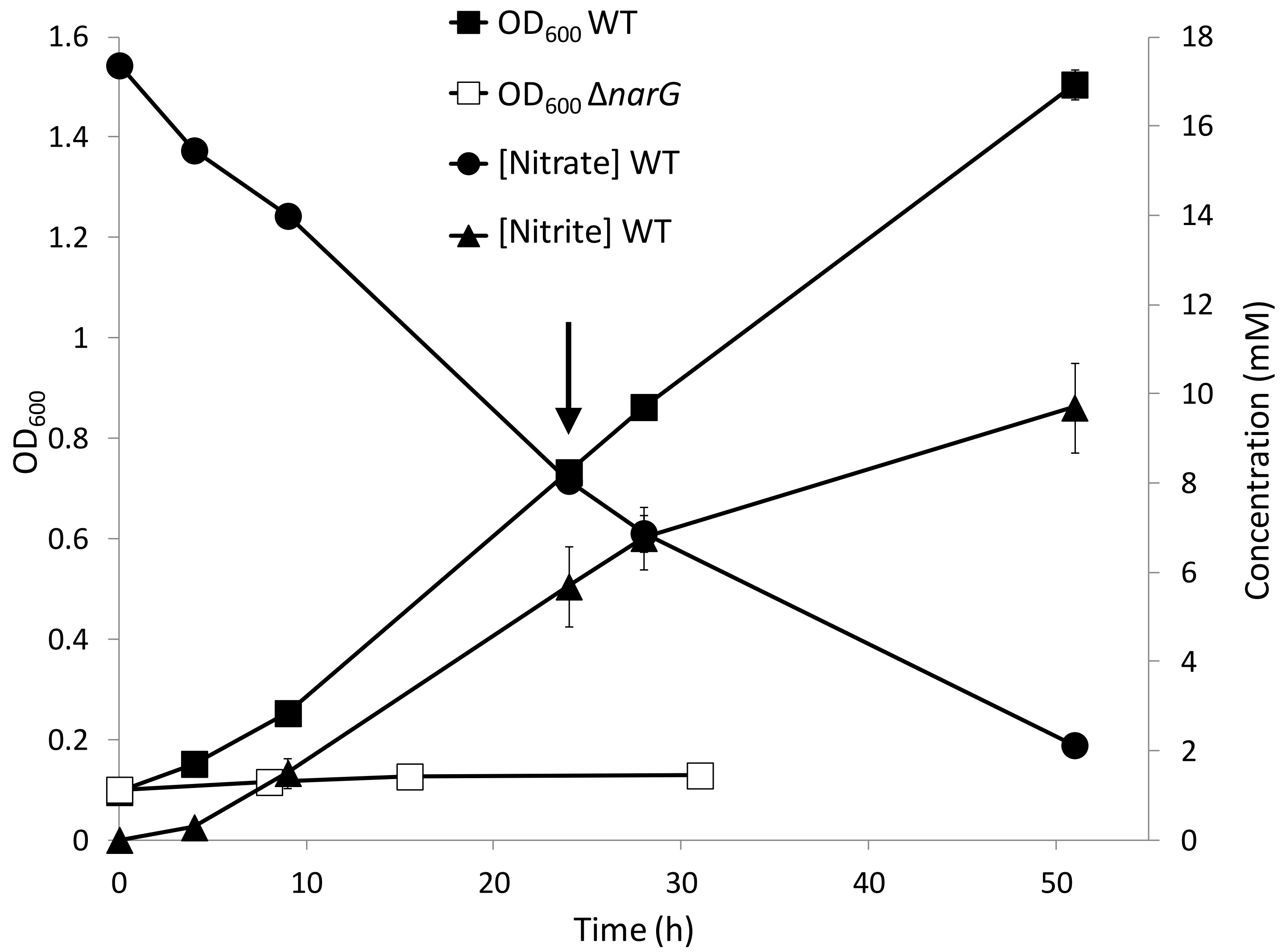

1 Garcia-Romero, I. *et al.* Genomic analysis of the nitrate-respiring *Sphingopyxis granuli* (formerly *Sphingomonas macrogoltabida*) strain TFA. *BMC Genomics* **17**, 93, doi:10.1186/s12864-016-2411-1 (2016).

2 Fischer, M., Alderson, J., van Keulen, G., White, J. & Sawers, R. G. The obligate aerobe *Streptomyces coelicolor* A3(2) synthesizes three active respiratory nitrate reductases. *Microbiology* **156**, 3166-3179, doi:10.1099/mic.0.042572-0 (2010).

3 Martinez-Perez, O., Lopez-Sanchez, A., Reyes-Ramirez, F., Floriano, B. & Santero, E. Integrated response to inducers by communication between a catabolic pathway and its regulatory system. *J Bacteriol* **189**, 3768-3775, doi:10.1128/JB.00057-07 (2007).

4 Yuste, L. *et al.* Growth phase-dependent expression of the *Pseudomonas putida* KT2440 transcriptional machinery analysed with a genome-wide DNA microarray. *Environ Microbiol* **8**, 165-177, doi:10.1111/j.1462-2920.2005.00890.x (2006).

5 Lindner, R. & Friedel, C. C. A comprehensive evaluation of alignment algorithms in the context of RNA-seq. *PLoS One* **7**, e52403, doi:10.1371/journal.pone.0052403 (2012).

6 Trapnell, C. *et al.* Transcript assembly and quantification by RNA-Seq reveals unannotated transcripts and isoform switching during cell differentiation. *Nat Biotechnol* **28**, 511-515, doi:10.1038/nbt.1621 (2010).

7 Gentleman, R. C. *et al.* Bioconductor: open software development for computational biology and bioinformatics. *Genome Biol* **5**, R80, doi:10.1186/gb-2004-5-10-r80 (2004).

8 Tatusov, R. L., Galperin, M. Y., Natale, D. A. & Koonin, E. V. The COG database: a tool for genome-scale analysis of protein functions and evolution. *Nucleic Acids Res* **28**, 33-36 (2000).
